# Paediatric antiretroviral update

**DOI:** 10.4102/sajhivmed.v24i1.1506

**Published:** 2023-07-06

**Authors:** Gillian Sorour, Nosisa Sipambo, Moherndran Archary

**Affiliations:** 1 Department of Paediatrics and Child Health, University of the Witwatersrand, Johannesburg, South Africa; 2 Discipline of Paediatrics and Child Health, College of Health Sciences, University of KwaZulu-Natal, Durban, South Africa; 3 Department of Paediatrics, King Edward VIII Hospital, Durban, South Africa

South Africa has lagged behind other countries in offering daily dosing of palatable, simple, well-tolerated and effective paediatric antiretroviral formulations to children with HIV.^1^ This is despite the availability of such formulations internationally, including our neighbouring countries in sub-Saharan Africa.

Since 2018, dolutegravir (DTG)-based antiretroviral treatment (ART) has been the preferred first-line treatment regimen in the World Health Organization guidelines for adults and children with HIV and has been adopted into South African guidelines for adults since November 2019.^2,3^ Dolutegravir is relatively inexpensive and has improved tolerability, fewer side effects and drug interactions and an increased genetic barrier to drug resistance when compared to other ART regimens.^4,5,6,7,8^ The inclusion of DTG into the guidelines for children and adolescents has been a much slower process. Based on a dosing sub-study of the Odyssey trial supporting the use of the DTG 50 mg tablet in children over 20 kg,^9^ South Africa’s 2019 ART guidelines expanded DTG access to children from 20 kg,^3^ which greatly improved treatment in these children. Further Odyssey sub-studies and the P1093 study also showed good data for DTG in children from 3 kg to 20 kg;^6,10,11^ however, for infants and children weighing less than 20 kg, access to DTG is dependent on availability of a new dispersible child-friendly formulation. First-line ART for infants and children < 20 kg has been a backbone of the protease inhibitor lopinavir/ritonavir and the two nucleoside-reverse-transcriptase inhibitors, abacavir and lamivudine. Lopinavir/ritonavir syrup needs to be given twice daily, should ideally be refrigerated, and is often poorly tolerated because of its awful taste. Abacavir and lamivudine were previously only available as relatively high-volume syrups for children unable to swallow tablets. Improvements came in the form of a 60 mg dispersible abacavir tablet and, finally, a fixed-dose combination scored dispersible tablet of abacavir and lamivudine (120/60 mg) which was approved in South Africa in 2021. The fixed-dose combination of abacavir and lamivudine can be administered as a once-daily dose and is easily dissolved in liquid for use in infants from 3 kg of weight.^12^ It is well tolerated and is an improvement on the large volumes of syrups that were previously required. Stock-outs and supply chain issues have hampered the roll-out of these formulations to all provinces and clinics.

South Africa’s recently released 2023 ART Clinical Guidelines for the Management of HIV^2^ come with many welcome improvements for infants and children living with HIV. The good news is that there are two new DTG formulations available: a scored 10 mg dispersible tablet that can be offered to infants from 4 weeks of age if they weigh at least 3 kg, and a single fixed-dose combination tablet of abacavir, lamivudine and DTG that can be given to children who weigh 25 kg or more.^12^ This aligns treatment with adults and will mean that a child will be able to be on DTG from diagnosis through infancy and childhood to adulthood. All infants and children who are currently on a non-DTG regimen, including second-line regimens, should now be evaluated for a switch to a DTG-based regimen.

Another change in the guidelines is that tenofovir can now be given to adolescents over 10 years who weigh at least 30 kg (previously 35 kg) and have normal kidney function.^2^ This means that adolescents over 10 years who weigh more than 30 kg can be transitioned to the fixed-dose combination of tenofovir/lamivudine/DTG in alignment with adult recommendations.

Countries in sub-Saharan Africa that have adopted the transition to paediatric DTG have demonstrated rapid uptake with improved viral suppression in children > 20 kg.^13^ Once-daily dosing of a more easily tolerated, effective medication will hopefully improve adherence and outcomes in our vulnerable paediatric population, including those < 20 kg, and support South Africa’s goal of achieving the last indicator in the Joint United Nations Programme on HIV/AIDS 95-95-95 initiative (diagnosis of 95% of all people living with HIV, initiating 95% on ART among those diagnosed and achieving 95% virally suppressed among those being treated). Current Joint United Nations Programme on HIV/AIDS treatment cascade figures in South African adults are 94-74-67, with children lagging far behind.^14^ In the paediatric population, only 83% of children living with HIV are known to be infected. Of these, only 48% are on treatment, and a mere 33% of those on treatment are virally suppressed.^14^ It is likely that the suboptimal regimens used in children in South Africa have played a large role in the poor viral suppression in this group.

The availability of more child-friendly formulations will undoubtedly be a relief to many parents and clinicians who have struggled with getting infants and children to tolerate existing formulations. The goal of a successful paediatric ART programme is to enable all children living with HIV to live a normal life at their full potential with a suppressed HIV viral load throughout their childhood and continued into adulthood and we hopefully will be moving closer to this goal with the switch to optimised regimens.

## References

[1] Archary M, Van Zyl R, Sipambo N, Sorour G. Optimised paediatric antiretroviral treatment to achieve the 95-95-95 goals. S Afr J HIV Med. 2021;22(1):1–4. 10.4102/sajhivmed.v22i1.1278 PMC8517773 34691769

[2] National Department of Health. 2023 ART clinical guidelines for the management of HIV in adults, pregnancy and breastfeeding, adolescents, children, infants and neonates. Pretoria: National Department of Health; 2023.

[3] National Department of Health. 2019 ART clinical guidelines for the management of HIV in adults, pregnancy, adolescents, children, infants and neonates. Pretoria: National Department of Health; 2019.

[4] Cevik M, Orkin C, Sax PE. Emergent resistance to dolutegravir among INSTI-naïve patients on first-line or second-line antiretroviral therapy: A review of published cases. Open Forum Infect Dis. 2020 Jun 2;7(6):ofaa202. 10.1093/ofid/ofaa202 32587877 PMC7304932

[5] Molina J-M, Clotet B, Van Lunzen J, et al. Once-daily dolutegravir versus darunavir plus ritonavir for treatment-naive adults with HIV-1 infection (FLAMINGO): 96 week results from a randomised, open-label, phase 3b study. Lancet HIV. 2015;2(4):e127–e136. 10.1016/S2352-3018(15)00027-2 26424673

[6] Turkova A, White E, Mujuru HA, et al. Dolutegravir as first-or second-line treatment for HIV-1 infection in children. N Engl J Med. 2021;385(27):2531–2543. 10.1056/NEJMoa2108793 34965338 PMC7614690

[7] Aboud M, Kaplan R, Lombaard J, et al. Dolutegravir versus ritonavir-boosted lopinavir both with dual nucleoside reverse transcriptase inhibitor therapy in adults with HIV-1 infection in whom first-line therapy has failed (DAWNING): An open-label, non-inferiority, phase 3b trial. Lancet Infect Dis. 2019;19(3):253–264. 10.1016/S1473-3099(19)30036-2 30732940

[8] Schramm B, Temfack E, Descamps D, et al. Viral suppression and HIV-1 drug resistance 1 year after pragmatic transitioning to dolutegravir first-line therapy in Malawi: A prospective cohort study. Lancet HIV. 2022;9(8):e544–e553. 10.1016/S2352-3018(22)00136-9 35905753

[9] Bollen PD, Moore CL, Mujuru HA, et al. Simplified dolutegravir dosing for children with HIV weighing 20 kg or more: Pharmacokinetic and safety substudies of the multicentre, randomised ODYSSEY trial. Lancet HIV. 2020;7(8):e533–e544. 10.1016/S2352-3018(20)30189-2 32763217 PMC7445428

[10] Ruel TD, Acosta EP, Liu JP, et al. Pharmacokinetics, safety, tolerability, and antiviral activity of dolutegravir dispersible tablets in infants and children with HIV-1 (IMPAACT P1093): Results of an open-label, phase 1–2 trial. Lancet HIV. 2022;9(5):e332–e340. 10.2139/ssrn.3954096 35489377 PMC9313528

[11] Waalewijn H, Chan MK, Bollen PD, et al. Dolutegravir dosing for children with HIV weighing less than 20 kg: Pharmacokinetic and safety substudies nested in the open-label, multicentre, randomised, non-inferiority ODYSSEY trial. Lancet HIV. 2022;9(5):e341–e352. 10.1016/S2352-3018(21)00292-7 35189082 PMC9046096

[12] Department of Health. Antiretroviral drug dosing chart for children 2022. Pretoria: National Department of Health; 2022.

[13] Bacha JM, Dlamini S, Anabwani F, et al. Realizing the Promise of Dolutegravir in effectively treating children and adolescents living with HIV in real-world settings in 6 countries in Eastern and Southern Africa. *The Pediatric Infectious Disease Journal* 42(7), 576–581. 10.1097/INF.0000000000003878 PMC10259212 36795586

[14] UNAIDS Data 2022. Joint United Nations Programme on HIV/AIDS (UNAIDS) [homepage on the Internet]. c2023 [cited 2023 May 31]. Available from: https://www.unaids.org/en/resources/documents/2023/2022_unaids_data

